# Artificial Intelligence–Enabled Analysis of Public Attitudes on Facebook and Twitter Toward COVID-19 Vaccines in the United Kingdom and the United States: Observational Study

**DOI:** 10.2196/26627

**Published:** 2021-04-05

**Authors:** Amir Hussain, Ahsen Tahir, Zain Hussain, Zakariya Sheikh, Mandar Gogate, Kia Dashtipour, Azhar Ali, Aziz Sheikh

**Affiliations:** 1 School of Computing Edinburgh Napier University Edinburgh United Kingdom; 2 Department of Electrical Engineering University of Engineering and Technology Lahore Pakistan; 3 Edinburgh Medical School College of Medicine and Veterinary Medicine University of Edinburgh Edinburgh United Kingdom; 4 NHS Forth Medical Group Grangemouth United Kingdom; 5 Harvard TH Chan School of Public Health Harvard University Boston, MA United States; 6 Usher Institute Edinburgh Medical School University of Edinburgh Edinburgh United Kingdom

**Keywords:** artificial intelligence, COVID-19, deep learning, Facebook, health informatics, natural language processing, public health, sentiment analysis, social media, Twitter, infodemiology, vaccination

## Abstract

**Background:**

Global efforts toward the development and deployment of a vaccine for COVID-19 are rapidly advancing. To achieve herd immunity, widespread administration of vaccines is required, which necessitates significant cooperation from the general public. As such, it is crucial that governments and public health agencies understand public sentiments toward vaccines, which can help guide educational campaigns and other targeted policy interventions.

**Objective:**

The aim of this study was to develop and apply an artificial intelligence–based approach to analyze public sentiments on social media in the United Kingdom and the United States toward COVID-19 vaccines to better understand the public attitude and concerns regarding COVID-19 vaccines.

**Methods:**

Over 300,000 social media posts related to COVID-19 vaccines were extracted, including 23,571 Facebook posts from the United Kingdom and 144,864 from the United States, along with 40,268 tweets from the United Kingdom and 98,385 from the United States from March 1 to November 22, 2020. We used natural language processing and deep learning–based techniques to predict average sentiments, sentiment trends, and topics of discussion. These factors were analyzed longitudinally and geospatially, and manual reading of randomly selected posts on points of interest helped identify underlying themes and validated insights from the analysis.

**Results:**

Overall averaged positive, negative, and neutral sentiments were at 58%, 22%, and 17% in the United Kingdom, compared to 56%, 24%, and 18% in the United States, respectively. Public optimism over vaccine development, effectiveness, and trials as well as concerns over their safety, economic viability, and corporation control were identified. We compared our findings to those of nationwide surveys in both countries and found them to correlate broadly.

**Conclusions:**

Artificial intelligence–enabled social media analysis should be considered for adoption by institutions and governments alongside surveys and other conventional methods of assessing public attitude. Such analyses could enable real-time assessment, at scale, of public confidence and trust in COVID-19 vaccines, help address the concerns of vaccine sceptics, and help develop more effective policies and communication strategies to maximize uptake.

## Introduction

The imminent availability of COVID-19 vaccines poses a pressing need to continually monitor and better understand public sentiments in order to develop baseline levels of confidence in them among the general public and enable the identification of early warning signals of loss in confidence [1]. This will help address the concerns of vaccine sceptics [2-4] and develop the required public trust in immunization [5,6] to realize the goal of generating herd immunity [7].

Traditionally, governments use surveys to understand public attitude; however, these typically have limitations including small sample sizes, closed questions, and limited spatiotemporal granularity. In order to overcome these limitations, we argue that social media data can be used to obtain more, real-time insights into public sentiments and attitudes with considerable spatiotemporal granularity. Over half of the worldwide population, including approximately 70% the populations of the United Kingdom and the United States, are active social media users, and social media usage has significantly increased during the pandemic; for instance, Facebook usage increased by 37%. Since social media data are largely unstructured, they are amenable to the application of established artificial intelligence (AI) techniques such as machine learning, deep learning (DL) [8], and natural language processing (NLP) [9] to extract topics and sentiments from social media posts.

Sentiment analysis involves categorizing subjective opinions from text, audio, and video sources [9] to determine polarities (eg, positive, negative, and neutral), emotions (eg, anger, sadness, and happiness), or states of mind (eg, interest vs disinterest) toward target topics, themes, or aspects of interest [10]. A complementary approach, termed stance detection [11], assigns a stance label (favorable, against, and none) to a post on a specific predetermined target, which in itself may not be referred to or be the target of opinion in the post. Such approaches are currently underutilized in health care research. In particular, there is significant untapped potential in drawing on AI-enabled social media analysis to inform public policy research.

## Methods

### Ethics

Since the data analyzed in this study were completely in the public domain, no ethics review was necessary. We conducted a thorough assessment of the privacy risk that our study posed to individuals, in accordance with previous reports [12,13], to ensure compliance with relevant sections of the General Data Protection Regulation. We strived to comply with best practices for user protection [14,15], ensuring that nonpublic material is not included in our data set. Further, to comply with privacy laws and social network policies in accordance with the General Data Protection Regulation to collect data from Twitter [16] and Facebook CrowdTangle [17] platforms, we have not shared or published direct tweets or posts by individuals, quotes from individuals, or names or locations of users who are not public organizations or entities.

### Data Sources

We used data from both Facebook and Twitter, two of the most popular and representative social media platforms [16]. We used Facebook posts and tweets that were posted in English in the United Kingdom and the United States from March 1 to November 22, 2020. Facebook posts were obtained through the CrowdTangle platform [17] and Twitter posts from a publicly available Twitter API. We used hydrated tweets from the global COVID-19 data set [18], which collects up to 4,400,000 tweets per day (including retweets) and up to 1,100,000 cleaned tweets without retweets. The total number of tweets hydrated and used in this study was >158,000,000. Facebook posts and tweets were thematically filtered for both COVID-19– and vaccine-related keywords and then geographically filtered for the United Kingdom and the United States. The first step in filtering COVID-19–related keywords involved widely used terms from the data set of Banda et al [18] (Multimedia Appendix 1). Vaccine-related terms used for second step filtering were selected by our team: “vaccine,” “vaccination,” “immunise,” “immunize,” “immunisation,” and “immunization.” A 2-step thematic filtering process was applied using these keywords before processing and analysis.

### Analysis

**Figure 1:**
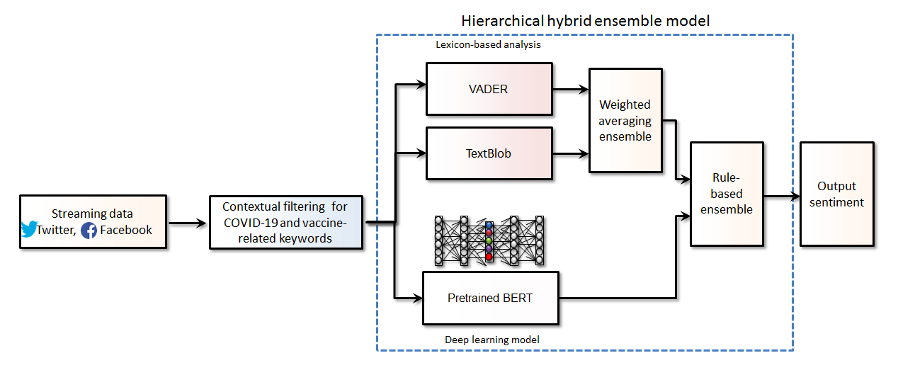
Hierarchical hybrid-ensemble–based artificial intelligence model and data pipeline for thematic sentiment analysis. BERT: Bidirectional Encoder Representations from Transformers, VADER: Valence Aware Dictionary for Sentiment Reasoning.

The filtered data set was initially preprocessed (eg, removing links, hashtags, and stop words) and a new hierarchical hybrid ensemble–based AI model was developed for thematic sentiment analysis. This utilized an average weighting ensemble [19] of 2 lexicon-based methods: Valence Aware Dictionary for Sentiment Reasoning (VADER) [20] and TextBlob [21]. These were combined with a pretrained DL-based model, Bidirectional Encoder Representations from Transformers (BERT) [22], using a rule-based ensemble method (Figure 1).

A random 10% sample of Facebook posts and tweets was then manually annotated by the team and screened against our hybrid ensemble AI model’s sentiment classifications for refinement and validation. The hybrid ensemble model was optimized on the basis of the validation results, with sensitivity and specificity analysis revealing that the lexicon-based methods provided generally better accuracy for positive sentiments, and the BERT model generally provided better accuracy for neutral and negative sentiments, as illustrated using normalized confusion matrices (Multimedia Appendix 1).

VADER and TextBlob were combined though weighted averaging (VADERx0.45 + TextBlobx0.55), with TextBlob assigned a marginally higher weight of 0.55 owing to its performance. The weighted averaged output from the lexicons was combined with the output of the BERT model, using a final rule–based ensemble. The final output was combined through “If” and “Else” statements on the basis of the model’s output sentiments.

A number of established NLP techniques were used to analyze the processed data (Multimedia Appendix 1). Specifically, in addition to analyzing averaged sentiment trends and their geospatial mappings in the United Kingdom and the United States, we statistically analyzed the trends with Pearson correlation coefficient (r) and compared the findings with those of independent surveys. Sentiment word cloud and N-gram analyses were performed for specific periods of interest, around points of inflexion on sentiment trend graphs, to identify topics of discussion and to gain insight into the positive and negative content of online discourses. The analysis was also carried out over the entire study period to identify underlying themes and topics. Findings were validated, and further insights were obtained through manual reading of randomly selected posts around target points of interest by our team. Relevant social media data sets and outputs were anonymized, and statistical aggregates were made openly accessible for transparency and reproducibility (additionally through a publicly available dashboard [23]).

## Results

### Temporal Sentiment Trends

**Figure 2:**
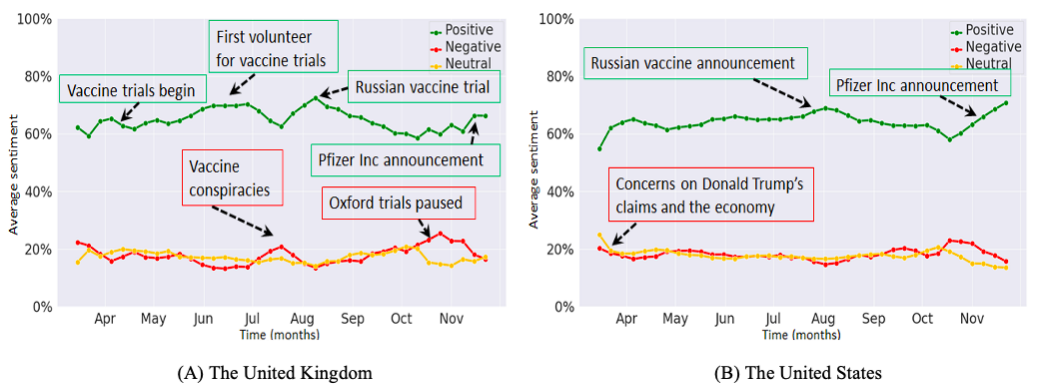
Averaged weekly trends in Facebook sentiments for (A) the United Kingdom and (B) the United States.

**Figure 3:**
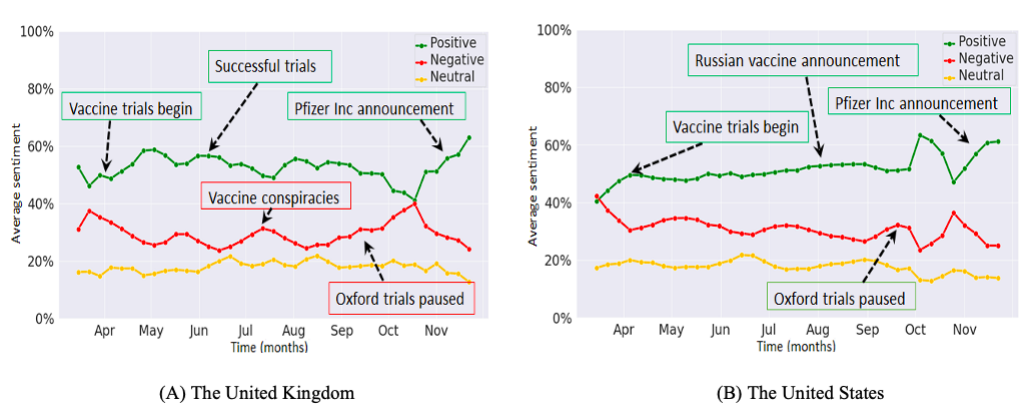
Average weekly trends in Twitter sentiments for (A) the United Kingdom and (B) the United States.

Monthly volume trends of the filtered Facebook posts and tweets in the United Kingdom and the United States for the target study period are shown in Multimedia Appendix 1. Figure 2 shows the averaged (weekly) positive, negative, and neutral Facebook sentiments in March-November 2020 in the United Kingdom and the United States. We identified topics of discussion on points of interest in the graphs. These are referred to in our descriptive analysis of the graphs below, and some are highlighted in Figures 2 and 3. It was interesting to note that the difference between the averaged positive and negative sentiment trends was more pronounced on Facebook than on Twitter.

For the United Kingdom, positive sentiments on Facebook displayed the most prominent trend (Figure 2A), showing a steady increasing trend since May 2020, corresponding to the initiation of vaccine trials and the recruitment of the first trial volunteer. We observed a peak in mid-August 2020, which was potentially associated with news on vaccine development in the United Kingdom and Russia. Negative sentiments on Facebook displayed an inverse trend to that of positive sentiments, and discourse was centered around vaccine conspiracies and halting of trials. For the United States, positive sentiments on Facebook displayed the most prominent trend, showing a small peak in August 2020, which was associated with posts relating to research on the COVID-19 vaccine in Russia. Moreover, negative sentiments on Facebook displayed a slight increase in mid-September 2020, which was associated with posts relating to the accelerated development of the COVID-19 vaccine (Figure 2B). More recently, from mid-October 2020, the trend of positive sentiments on Facebook in the United Kingdom and the United states increased, partly because of announcements from Pfizer Inc and Moderna Inc on successful vaccine trials [24].

Figure 3 illustrates the positive, negative, and neutral sentiments on Twitter from March to November 2020 for the United Kingdom and the United States. Figure 3A shows that in the United Kingdom, positive sentiments on Twitter displayed the most prominent trend, showing a small peak at the end of April and July of 2020, the former related to the first human vaccine trial. The negative sentiment trend on Twitter displayed peaks in July and October of 2020, simultaneously with the United Kingdom opting out of the European Union vaccination scheme and halting of the phase III vaccine trials at the University of Oxford owing to safety concerns [25]. Figure 3B shows that in the United States, positive sentiments on Twitter displayed the most prominent trend, showing major peaks from end-September to end-November of 2020, which was related to claims by ex-President Donald Trump regarding a vaccine being ready in a few weeks and an increase in Twitter discourse due to his reference to the “herd mentality.” We observed a small peak in the negative trend graph in mid-September 2020, which was related to halting of the phase III vaccine trial at the University of Oxford.

For both the United Kingdom and the United States, we observed a marked increase in the positive sentiment trend, since end-October 2020, which was related to recent breakthrough announcements by Pfizer Inc and Moderna Inc. Analysis of social media conversations indicated public optimism, with trial results being hailed as “good” and “amazing” and with “hope” prevailing for the “new year” (Multimedia Appendix 1). A notable peak in the negative sentiment trends for both countries, in approximately mid-October 2020, was associated with the growing antivaccination movement and with concerns regarding “fake news” and “misinformation.”

### Statistical Analysis of Sentiment Trends

Statistical analysis (results detailed in Multimedia Appendix 1) involved the assessment of the strength of the association between the predicted sentiment in the trend graph and the accuracy of the labeled data. Overall, regarding COVID-19 vaccines, we observed stronger sentiments on Twitter for the United States, with both positive and negative sentiments displaying stronger increasing and decreasing trends, respectively, compared to the United Kingdom. Public sentiments on Facebook reflected a reduction in positive sentiments and an increase in neutral sentiments in both the United Kingdom and the United States, with positive sentiments displaying a slightly stronger decreasing trend in the United Kingdom than in the United States.

### Sentiment Word Clouds and Text N-Gram Analysis 

We performed sentiment word cloud and text N-gram analyses for the entire study period to identify and analyze notable events that were of interest to social media users, and the findings are summarized in Multimedia Appendix 1 (some of these were also identified in the aforementioned analysis, on the sentiment trend graphs).

### Geospatial Sentiment Analysis

**Figure 4:**
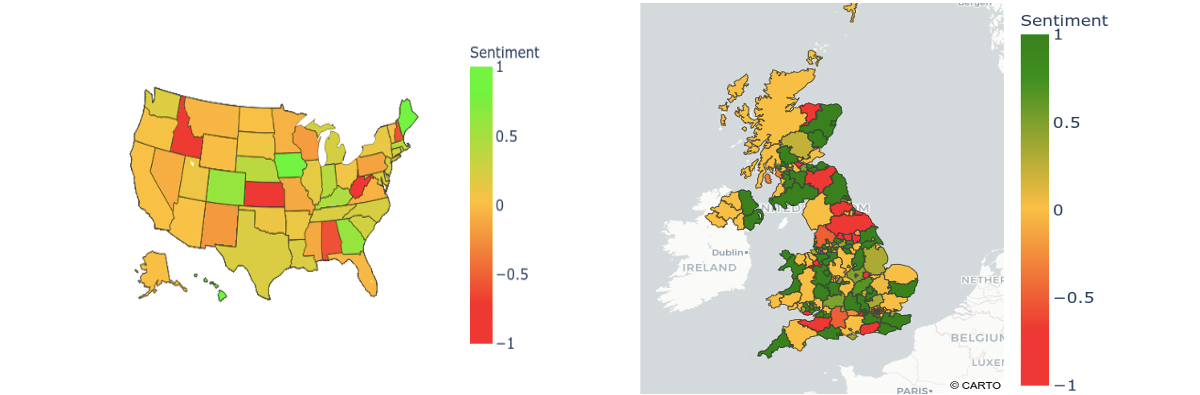
Geospatial mapping of averaged social media public sentiments in the United States (left) and the United Kingdom (right) toward COVID-19 vaccines (1 or green: positive sentiments, 0: neutral sentiments, and -1 or red: negative sentiments).

A geospatial map of overall (averaged) sentiments at the state level in the United States is shown in Figure 4 (left), and it indicates that most states had a negative sentiment. The states with an overall negative sentiment toward COVID-19 vaccines were concentrated in the West and Midwest regions, namely Idaho, Kansas, New Hampshire, West Virginia, and Alabama. The states with an overall positive sentiment were in the East, namely Maine, Colorado, Georgia, and Hawaii.

A geospatial map of averaged sentiments toward COVID-19 vaccines at the county level in the United Kingdom is shown in Figure 4 (right). In contrast with the United States, most counties in the United Kingdom had an overall positive sentiment toward COVID-19 vaccines. The counties with the most positive sentiments included Cornwall, Kent, East Sussex, Surrey, and Dorset in England and Aberdeenshire, Angus, and Stirlingshire in Scotland. Furthermore, the counties with the most negative sentiments were West Sussex, Somerset, North Yorkshire, and Durham in England.

### Overall Averaged Sentiments

**Figure 5:**
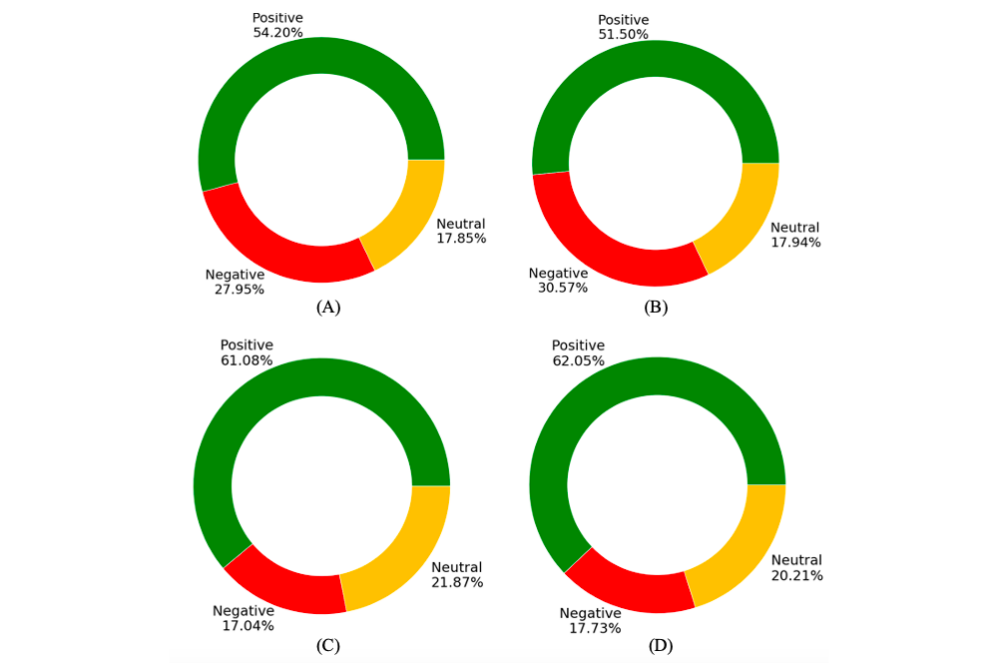
Overall averaged sentiments: (A) Twitter sentiments in the United Kingdom, (B) Twitter sentiments in the United States, (C) Facebook sentiments in the United Kingdom, and (D) Facebook sentiments in the United States.

Overall averaged sentiments in the United Kingdom and the United States on Facebook and Twitter are shown in Figure 5 and in Multimedia Appendix 1.

## Discussion

### Principal Findings

We analyzed temporal variations in public sentiments toward COVID-19 vaccines in the United Kingdom and the United States. We identified, evaluated, and mapped the key events impacting positive, negative, and neutral sentiments to the temporal trends. We also mapped spatial variations in public sentiment to regions in the United Kingdom and states in the United States. Our geospatial maps can help identify areas with more negative sentiments toward COVID-19 vaccines, which can be further studied for potential interventions, to allay the underlying public fears and concerns.

Our findings indicate that online public discourse on Facebook and Twitter across the United Kingdom and the United States is evolving, with both complementary and contrasting insights obtained from the 2 popular platforms. Comparative analysis revealed that over the 9-month study period, averaged public sentiment toward COVID-19 vaccines has been mostly positive and similar in both the United Kingdom and the United States across both platforms (57.70% average across both platforms for the United Kingdom vs 56.80% for the United States). Positive sentiments were related to public opinions on vaccine development, related trials, and news related to vaccine availability. 

On both platforms, overall averaged negative sentiments were found to be similar for the United Kingdom (22.50%) and the United States (24.10%). It is interesting to note that Twitter sentiments appeared more negatively biased, with the proportion of negative sentiments being almost 2-fold those on Facebook, for both the United Kingdom (27.95% vs 17.04%) and the United States (30.57% vs 17.73%), which potentially reflects their respective user demographics. This finding appears consistent with those of Waterloo et al [16], who reported that public opinions were often more negatively biased on Twitter than on Facebook, with public opinions being more positively biased on Facebook. Negative sentiments in our study were related to public apprehensions and concerns regarding delays or pauses in vaccine trials, vaccine safety, corporations, and governments influencing vaccine availability and rights exclusivity for economic benefits.

A comparative analysis with independent surveys was carried out. Our findings related to trends of averaged positive and negative sentiment across the United Kingdom and the United States were found to correlate broadly. In the United States, during the early stages of the pandemic, polling indicated that a significant minority had low trust in a vaccine; for example, a Yahoo News/YouGov survey in May 2020 [26] reported that only 55% of people in the United States intended to get vaccinated against COVID-19, while almost 1 in 5 (19%) individuals would not get vaccinated. A similar survey in July 2020 [27] reported that 42% of people in the United States would get vaccinated (27% would not get vaccinated), while a survey in September 2020 [28] reported that only 36% of people in the United States were certain they would get vaccinated (32% would not get vaccinated). More recently, an Axios-Ipsos survey in November 2020 reported, consistent with the findings of our social media analysis, a marked increase in the proportion of individuals who are likely to get vaccinated (51% were “very” or “somewhat” likely to take the first-generation vaccine; this proportion would increase to 70% if the vaccine was proven safe and effective by public health officials) [29].

In the United Kingdom, a YouGov survey in June 2020 [30] reported that 41% of respondents would “probably” or “definitely” get vaccinated, while 1 in 6 (16%) respondents would “definitely” or “probably” not get vaccinated. The survey also reported that individuals who used social media more than traditional media as their source of news were 9% less likely to be in favor of being vaccinated. A more recent YouGov survey in the United Kingdom in November 2020 [31], related to the Pfizer COVID-19 vaccine, reported that 67% of individuals were “very” or “fairly” likely to take the vaccine when available, and approximately 1 in 5 (21%) individuals were unlikely to take it. While there has been a slight increase in the proportion of individuals unlikely to take the vaccine, the proportion of those likely to get vaccinated has increased, indicating a reduction in the number of individuals who were previously unsure. This could be attributed in part to the recent announcements by vaccine manufacturers, and our results corroborate this finding with a marked increase in positive sentiments since mid-October 2020, in both the United Kingdom and the United States, across the 2 social media platforms. Further studies on changes in sentiments could further the current understanding of factors that have contributed to this, with particular focus on the impact of government education programs.

### Limitations

It is important to consider the limitations of our data sources and techniques and the related challenges and opportunities they present for future research. While we attempted to gauge nationwide public sentiments in the United Kingdom and the United States by analyzing posts in English on both Facebook and Twitter, our data may not be representative of the broader population of both countries. Users are known to differ in their usage and preferences regarding social media platforms on the basis of their sociodemographics (eg, age, socioeconomic status, and political affiliation). Vaccines are likely to be preferentially targeted at older populations and possibly ethnic minorities, communities with historically lower rates of vaccine uptake [32,33]. Further exploration is therefore imperative to increase our understanding of the public perception toward vaccines and their underlying behavioral determinants [34]. Social network analysis [35,36] can be performed in conjunction with DL methods to effectively identify sources of fake news or misinformation and their social networks to help deal with infodemic challenges [37,38]. Demographic data including age, gender, race, and geographic origin can also be inferred from social media profiles of users by using AI techniques [39]. This can help categorize distinct groups and inform the development of demographic-level engagement and tailored communication strategies to promote diversity and inclusion in vaccination campaigns. These can also effectively account for the fact that there are genuine knowledge voids being filled by misinformation [34].

The technical limitations of our approach include challenges in determining the geographic location of users and issues relating to the accuracy of the AI techniques (eg, interpreting sarcasm and implicit context). Alternative deep neural networks [40-42] and fuzzy-based approaches [43,44] can be explored and potentially integrated as part of our ensemble model in an attempt to further refine the present findings. The 2-step keyword-based thematic filtering process and the use of geotagged posts in this study resulted in relatively small sample sizes. This could be improved by using more sensitive filtering and data-driven search mechanisms, network metadata (such as likes and retweets), and additional social media and web-based platforms. On account of the current study limitations, our approach should only be used in conjunction with other techniques for understanding public sentiments, such as focus groups, input from civil society organizations, surveys, and public consultations.

Future studies could consider conducting periodic public surveys over the period of interest being explored through social media analysis. This would ensure that both methodologies were informed by each other over the course of the study to enable more granular spatiotemporal analysis, thus allowing more robust comparisons from reciprocal findings and deeper insights for policymakers. These could also complement other qualitative methods, such as in-depth interviews and ethnographic studies, as part of mixed-study approaches. Manual annotation or labeling of datasets is imperative when training AI models for NLP tasks to ensure accuracy and generalizability. These can be affected by the skill of annotators and the proportion of the data set that is labeled. Confounding factors, such as political affiliations, should also be included in future studies, by applying further filters to screen strategies and through targeted demographic analysis, to further the current understanding of the underlying determinants of public sentiments. Attitudes toward different vaccine manufacturers could also be explored to identify and assess effective public engagement strategies to build support for ethical principles and maximize the uptake of the imminently available vaccines.

### Conclusions

One of the main threats to the resilience of vaccination programs globally is the rapid and global spread of misinformation. Public confidence in COVID-19 vaccines can be exacerbated by unproven concerns regarding vaccine safety, which seed doubt and distrust. Furthermore, there have been cases where vaccine debates have been purposefully polarized, thus exploiting the doubting public and system weaknesses for political purposes, while waning vaccine confidence elsewhere may be influenced by a general distrust in the government and scientific elites. Recent surveys and polls in the United Kingdom and the United States have indicated the fragility of support for vaccination, which furthers the requirement for a better understanding of underlying public concerns and attitudes, both at scale and in real time. Retrospective analysis of 2 popular and most representative social media platforms in this study demonstrates the potential of AI-enabled real-time social media monitoring of public sentiments and attitudes to help detect and prevent such fears and also to enable policymakers to understand the reasons why some social groups may be reluctant to be vaccinated against COVID-19. This can inform more effective policy-making and promote participatory dialogue on complex vaccine deployment issues, under conditions of uncertainty, including decisions on prioritization and equitability, to help maximize the uptake of imminently available vaccines.

## Appendix

Multimedia Appendix 1 Supplementary Material as quoted in the main article.

## Abbreviations

AI: artificial intelligence
BERT: Bidirectional Encoder Representations from Transformers
DL: deep learning
NLP: natural language processing
VADER: Valence Aware Dictionary for Sentiment Reasoning
